# Functional Diversification of *euANT/PLT* Genes in *Oryza sativa* Panicle Architecture Determination

**DOI:** 10.3389/fpls.2021.692955

**Published:** 2021-07-09

**Authors:** Ai My Luong, Hélène Adam, Carole Gauron, Pablo Affortit, Fabrice Ntakirutimana, Ngan Giang Khong, Quang Hoa Le, Thi Nhu Le, Marie Fournel, Michel Lebrun, James Tregear, Stefan Jouannic

**Affiliations:** ^1^DIADE, University of Montpellier, IRD, CIRAD, Montpellier, France; ^2^LMI RICE, National Key Laboratory for Plant CellBiotechnology, Agronomical Genetics Institute, University of Montpellier, IRD, CIRAD, University of Science and Technologyof Hanoi, Hanoi, Vietnam; ^3^School of Biotechnology and Food Technology, Hanoi University of Science and Technology, Hanoi, Vietnam; ^4^LSTM, University of Montpellier, IRD, CIRAD, INRAE, SupAgro, Montpellier, France

**Keywords:** panicle, branching, meristem, PLETHORA, rice, yield

## Abstract

Grain yield, which is one of the most important traits in rice breeding, is controlled in part by panicle branching patterns. Numerous genes involved in the control of panicle architecture have been identified through mutant and QTL characterization. Previous studies suggested the importance of several AP2/ERF transcription factor-encoding genes in the control of panicle development, including the *AINTEGUMENTA/PLETHORA-like* (*euANT/PLT*) genes. The *ANT* gene was specifically considered to be a key regulator of shoot and floral development in *Arabidopsis thaliana*. However, the likely importance of paralogous *euANT/PLT* genes in the regulation of meristem identities and activities during panicle architecture development has not to date been fully addressed in rice. In this study, we observed that the rice *euANT/PLT* genes displayed divergent temporal expression patterns during the branching stages of early panicle development, with spatial localization of expression in meristems for two of these genes. Moreover, a functional analysis of rice *ANT*-related genes using genome editing revealed their importance in the control of panicle architecture, through the regulation of axillary meristem (AM) establishment and meristem fate transition. Our study suggests that the paralogous *euANT/PLT* genes have become partially diversified in their functions, with certain opposing effects, since they arose from ancestral gene duplication events, and that they act in regulating the branching of the rice panicle.

## Introduction

Rice is a staple food for over half of the population in the world, and around one billion people depend economically on rice cultivation. A sustainable increase in rice production against a backdrop of climate change, diminishing water, and land availability requires the plant to have an improved grain output, which needs to be doubled by 2050 (McClung, 2014). One of the most important morphological traits that contributes to rice yield potential is the architecture of the inflorescence (or panicle), as the number of grains per panicle is determined by the total number of spikelets produced on the higher-order branches (Ikeda et al., 2004; Xing and Zhang, 2010; Tanaka et al., 2013).

The architecture of the mature inflorescence is initially determined during the reproductive phase, through meristematic activities that define the branching pattern along with flower positioning (Xing and Zhang, 2010; Kyozuka et al., 2014). Rice panicle development is governed by the activities of four different types of inflorescence meristems: rachis, branch, spikelet, and floral meristems. At the onset of reproductive development, the shoot apical meristem (SAM) is converted into a rachis meristem (RM). Subsequently, axillary meristems (AMs) are generated to form primary branch meristems (PBMs). During the elongation of primary branches (ePBM), the PBMs generate AMs, which may develop into secondary and higher-order branches or be transformed into lateral spikelet meristems (SpMs). Both PBMs and secondary branch meristems (SBMs) eventually form a terminal SpM. The RM, PBM, and ePBM/AM stages are indeterminate (i.e., meristematic cells are maintained), whereas the SpM is determinate in that the stem cells lose their activity (Ikeda et al., 2004; Tanaka et al., 2013; Kyozuka et al., 2014).

Our previous studies used comparative genome-wide expression profiling either between the different reproductive meristem types in the *Oryza sativa* panicle or between four distinct *Oryza* species at the branching stage of the panicle development. The results obtained revealed a large number of *APETALA2/Ethylene Responsive Factor* (*AP2/ERF*) genes displaying differential expression patterns between the different reproductive meristems and/or between different species (Harrop et al., 2016, 2019), suggesting roles in rice panicle architecture development and the determination of its diversity across wild and domesticated species. AP2/ERF transcription factors are a family of proteins defined by a conserved domain containing about 60–70 amino acids. They can be subdivided into four subfamilies (AP2, ERF, DREB, and RAV) depending on the number of AP2/ERF domains in the protein and the existence of other DNA binding domains (Nakano et al., 2006; Sharoni et al., 2011; Rashid et al., 2012). The AP2 subfamily includes members characterized by a tandem repetition of two AP2 domains plus a small number of proteins containing a single AP2 domain (Licausi et al., 2013) and is differentiated into three groups: *euAP2*, *euANT/PLT*, and *basalANT*. In contrast to the *euAP2* genes, the *basalANT* (or *WRINKLED*-like) genes and the *euANT/PLT* (or *AINTEGUMENTA-LIKE/PLETHORA*) genes do not possess a *miR172* recognition site and are distinguished by specific amino acid signatures (Kim et al., 2005; Dipp-Álvarez and Cruz-Ramírez, 2019).

There is evidence that certain AP2 subfamily genes are involved in panicle meristem identity determination by regulating the expression of reproductive meristem identity genes, notably in crop species. For example, the *euAP2* genes *SUPERNUMERARY BRACT* (*SNB*) and *OsINDETERMINATE SPIKELET 1* (*OsIDS1*) genes from rice, as well as *INDETERMINATE SPIKELET 1* (*IDS1*) from maize, *SCHLAFMUTZE* (*SMZ*), *SCHNARCHZAPFEN* (*SNZ*), and *TARGET OF EAT1* (*TOE*) genes from *Arabidopsis thaliana*, have been properly studied in the context of their role in floral ontogeny, such as in floral meristem identity and flowering time (Chuck et al., 1998; Lee and An, 2012; Zhang et al., 2015; Wang et al., 2016; Huang et al., 2017; Gras et al., 2018). Both *osids1* and *snb* mutants in rice showed a significant decrease in branch and spikelet numbers within a panicle (Lee and An, 2012). Moreover, *IDS1* orthologs in wheat (*Q*) and barley (*INTERMEDIUM-M* or *HvAP2L-H5*) control inflorescence structure and restrict the floret number per spikelet (Houston et al., 2013; Skov Kristensen et al., 2016; Greenwood et al., 2017; Gauley and Boden, 2019; Zhong et al., 2021). In *A. thaliana*, the *basalANT* group has been functionally described to play a role in fatty acid and cuticular wax biosynthesis (To et al., 2012; Park et al., 2016). In contrast, studies on rice revealed that the *WRINKLED1* ortholog (*OsWRI1*) was ubiquitously expressed in developing rice seeds (Mano et al., 2019), while *SMALL ORGAN SIZE1* (*SMOS1* or *OsERF142)* was shown to control organ size, including that of the panicle, and to modulate the root meristem size (Aya et al., 2014; Li et al., 2015; Hirano et al., 2017).

The euANT/PLT proteins are well known for their involvement in determining the stem cell fate, in the promotion of organ growth, and in the suppression of differentiation in the context of shoot meristem function in *A. thaliana* (Horstman et al., 2014; Scheres and Krizek, 2018; Krizek et al., 2020). The *AINTEGUMENTA* (*ANT*) gene of *A. thaliana* was considered to be the main regulator of shoot development within the *euANT/PLT* group of genes found in this species (Scheres and Krizek, 2018). Moreover, the *A. thaliana AINTEGUMENTA-like 5, 6*, and *7* (*AIL5*, *AIL6*, and *AIL7*) genes were all observed to be expressed in developing flowers and to have partly overlapping functions with the *ANT* gene in flower development (Krizek, 2015; Scheres and Krizek, 2018; Krizek et al., 2020). The *SvANT1* gene from millet *Setaria viridis* was reported to impact the tiller number, panicle number, and grain yield in association with a negative effect on the photosynthesis efficiency and growth rate (Liu et al., 2020). In expression studies performed on domesticated and wild rice species, the *euANT/PLT*-related genes *OsPLT9* and *OsPLT10* were identified as genes associated with panicle branching (Harrop et al., 2019). The *CROWN ROOTLESS 5* (*CRL5/OsPLT8*) gene was also demonstrated to be induced by auxin and involved in the *de novo* pathway to initiate crown roots (CRs) in rice, as well as being highly expressed in the early stages of inflorescence development (Kitomi et al., 2011). However, the roles of these *ANT*-related genes in rice panicle development remain to be addressed. In this study, we report on the characterization and expression analysis of rice *euANT*/*PLT* genes and, more specifically, the *ANT*-homologous genes. We describe the detailed phenotyping of CRISPR-Cas9 edited mutants of these genes, shedding light on their involvement in the regulation of panicle structure through their activities in the different reproductive meristems.

## Materials and Methods

### Plant Materials and Growth Conditions

Plants were grown in the greenhouse at IRD Montpellier (France), under long day conditions (14-h light/10-h dark) at 28–26°C and 60% humidity. After 6–8 weeks, flowering was induced under a cycle of 10 h of daylight. Panicles were collected at different stages according to the experiments. For *in situ* hybridization samples and single-panicle Fluidigm qRT-PCR experiments, young *O. sativa ssp. japonica* cv. Nipponbare panicles were collected. The *crl5* mutant and associated wild-type line (*O. sativa ssp. japonica* cv. Kinmaze) were kindly provided by Prof. Yoshiaki Inukai of Nagoya University (Kitomi et al., 2011). CRISPR-Cas9 edited plants were generated from *O. sativa ssp. japonica* cv. Kitaake.

### RNA *in situ* Hybridization

Panicle samples were collected from the main stem at different developmental stages and were embedded in Paraplast X-TRA (Sigma-Aldrich) as described by Huijser et al. (1992). Digoxigenin-labeled antisense and sense RNA probes were generated with the DIG RNA Labeling Kit SP6/T7 (Roche) according to the manufacturer’s instructions using primers containing a T7 promoter. The primers used to generate the probes are listed in Supplementary Table 1. Hybridization was performed as described in the study by Adam et al. (2011) using a VECTOR Blue Alkaline Phosphatase Substrate Kit (Vector Laboratories) for detection.

### RNA Extraction and Fluidigm^®^ High-Throughput qPCR

For the expression profiling during early panicle development in *O. sativa* cv. Nipponbare, a single panicle per stage and per biological replicate was collected as described in the study by Khong et al. (2021): N1, RM; N2, primary branch (PB) initiation; N3, early PB elongation; N4, PB elongation with AMs; N5, SpM differentiation; N6, floret meristem differentiation; and N7, floral organ establishment. Total RNA was extracted using an RNeasy Micro kit (Qiagen) from three biological replicates. High-throughput qRT-PCRs using a Biomark HD Microfluidic 96 × 96 Dynamic Array (Fluidigm^®^) were carried out as described in the study by Harrop et al. (2019). Gene expression relative to normalization standards was calculated using the normalized relative quantification method with multiple housekeeping genes (Pfaffl, 2001; Pfaffl et al., 2002). Primer sequences are listed in Supplementary Table 1.

### *In silico* Identification of *euANT/PLT* Genes in *A. thaliana* and Rice Genomes

In order to perform an exhaustive analysis of *euANT/PLT* genes from *O. sativa* and *A. thaliana*, AP2 subfamily genomic and protein sequences were retrieved from the TAIR database for *A. thaliana* (Berardini et al., 2015) and from the databases MSU (MSU7.0, Kawahara et al., 2013), RAPdb (IRGSP−1.0, Sakai et al., 2013), and NCBI (annotation version 102)^1^ for *O. sativa ssp. japonica*. Protein alignments and phylogeny trees were built using MEGA 7.0 to determine the relationships between the *euANT/PLT* genes in these rice species. Due to inconsistencies observed between the three databases for *O. sativa ssp. japonica euANT/PLT* gene annotations (Supplementary Table 2), we sequenced PCR fragments from several cDNAs obtained from *O. sativa ssp. japonica* cv. Nipponbare and subsequently opted to base our analyses on NCBI annotations. Combined unrooted neighbor-joining (NJ) trees were generated using MEGA 7.0 with the following default parameters: p-distance correction, pairwise deletion, and bootstrap (1,000 replicates). The visual phylogeny tree was built using EvolView version 3.0^2^ (Subramanian et al., 2019).

### Generation and Selection of CRISPR-Cas9 Edited Plants

CRISPR-Cas9 plasmid vector constructions were generated using the polycistronic gRNA-tRNA plasmid system according to the study described by Xie et al. (2015). Two 20 nt gRNAs were designed per gene to target the second AP2 domain-encoding region of the *euANT/PLT*-targeted genes according to available sequences from the three databases for *O. sativa ssp. japonica* cv Nipponbare (i.e., MSU, RAPdb, and NCBI) and based on sequences in the Phytozome v12 database (Goodstein et al., 2012) for the variety *O. sativa ssp. japonica* cv. Kitaake used for genetic transformation (see Supplementary Table 1 for the primer list). The pRGEB32 plasmid-derived constructs were transformed into *Agrobacterium tumefaciens* strain EHA105. Primers used for constructions generated with this vector are listed in Supplementary Table 1. *O. sativa ssp. japonica* cv. Kitaake plants were genetically transformed as previously described (Sallaud et al., 2003). CRISPR-Cas9-induced deletions in the first-generation regenerated plants (T0) were detected by PCR (see Supplementary Table 1 for primers) and sequence analysis using DSDecode and CRISP-ID web-based tools (Liu et al., 2015; Dehairs et al., 2016). T1 Cas9-free plants homozygous for the deletion were selected by PCR using primers recognizing the *HptII* and *Cas9* genes (see Supplementary Table 1 for primers) for further analysis, along with T1 Cas9-free plants without the mutation designated as sister plants.

### Plant Phenotyping

For the *crl5* mutant and associated wild-type genetic backgrounds (*O. sativa ssp. japonica* cv. Kinmaze), as well as for the T2 generation of homozygous CRISPR-Cas9 induced mutant lines from *O. sativa* cv. Kitaake and associated sister lines, at least 18 mature panicles of each line (i.e., three main panicles from at least six plants per line) were used for panicle phenotyping. For phenotyping analyses, each panicle was spread out on white paper and photographed. The images were then used for panicle structure and seed number analysis using P-TRAP software (AL-Tam et al., 2013). Morphological traits of the panicles measured using P-TRAP were rachis length (RL); number of spikelets per panicle (SpN); number of PB, SB, or tertiary branches per panicle (PBN, SBN, and TBN); length of PB or SBs per panicle (PBL and SBL); and lengths of the associated internodes (PBintL and SBintL) (Supplementary Figure 1). Subsequently, determination of the number of AMs per PB (AM) and of the ratio of secondary branch (SB) number vs. number of AMs per PB (SBN.AM) based on P-TRAP outputs was carried out, and data were analyzed according to the study described by Agata et al. (2020) using normalized PB position in the panicle. Other trait values were recorded during the development of the plants, including flowering time, plant height, the number of tillers, and the number of efficient tillers (i.e., tiller producing a panicle) per plant.

## Results

### Expression Diversity of *euANT/PLT* Genes During Panicle Development

**FIGURE 1:**
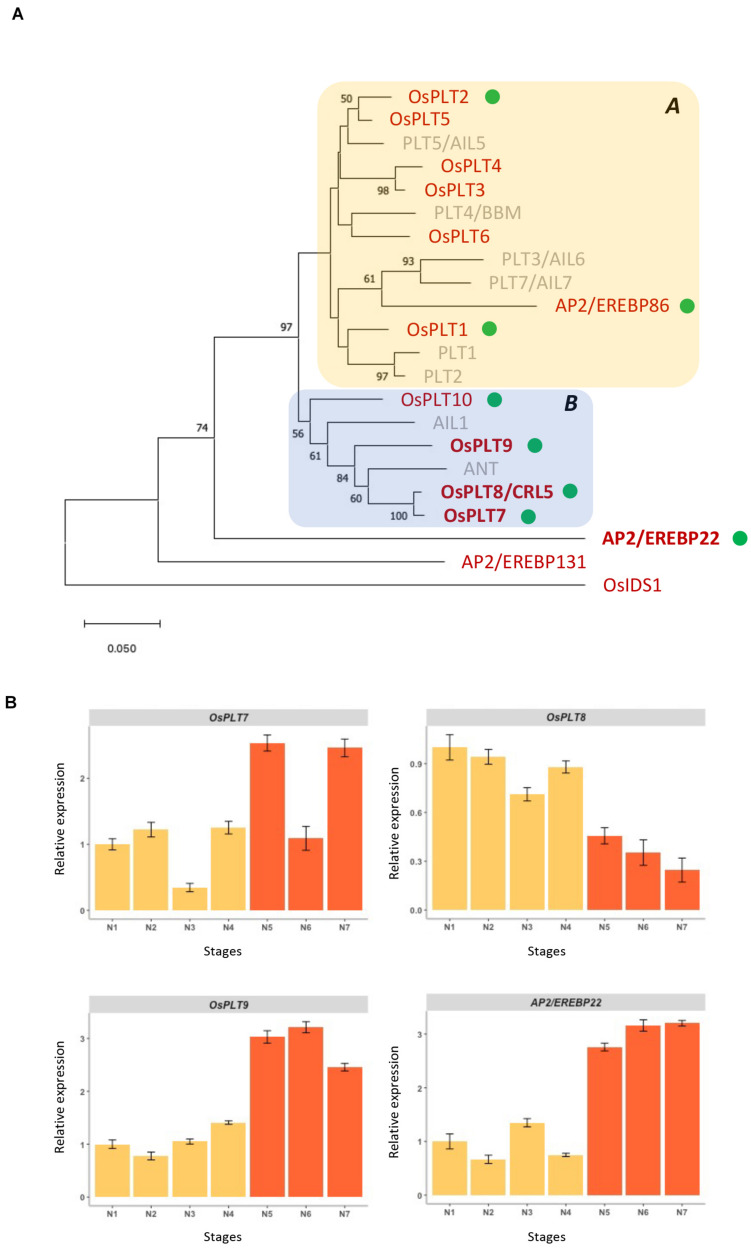
Phylogenetic relationships of *O. sativa* and *A. thaliana euANT/PLT* genes and expression patterns of *O. sativa ANT* homologs during panicle development. **(A)** Phylogenetic tree of the deduced amino acid sequences comprising the two AP2 domains and the linker of the euANT/PLT proteins from *O. sativa* cv. Nipponbare (red) and *A. thaliana* (gray) using the NJ method. Bootstrap values (1,000 tests), when equal to or greater than 50%, are indicated at individual nodes. Members of clades A and B, as defined by Li and Xue (2011), are indicated by colored boxes. *O. sativa* genes for which transcripts were detected in the young panicle *via* RNAseq (Harrop et al., 2016, 2019) are indicated by green dots. *O. sativa* genes selected for further analysis during the panicle development are indicated in boldface. AP2-EREBP131 and OsIDS1 were used as outgroups, as members of the *basalANT* and *euAP2* groups, respectively. **(B)** Expression analysis of the *O. sativa ANT* homologs and *AP2/EREBP22* gene during the panicle development using qRT-PCR. N1, rachis meristem; N2, primary branch initiation; N3, early primary branch elongation; N4, primary branch elongation with axillary meristems; N5, spikelet meristem differentiation; N6, floret meristem differentiation; N7, floral organ establishment. Stages N1 to N4 correspond to indeterminate stages (yellow bars), while stages N5–N7 to determinate stages (orange bars). Data are given as means ± SD from three independent biological replicates.

In *O. sativa ssp. japonica*, the AP2 subfamily consists of 27 genes divided into three groups, namely, the *euAP2*, *basalANT*, and *euANT/PLT* groups, in comparison with *A. thaliana* with 18 genes in total. In the *euANT/PLT* group, ten *PLETHORA* (*PLT*) genes were previously reported in *O. sativa ssp. japonica* [*OsPLT1* to *OsPLT10* (Li and Xue, 2011)] compared to eight members of the *euANT/PLT* group in *A. thaliana* (Mähönen et al., 2014; Scheres and Krizek, 2018; Figure 1A and Supplementary Table 2). According to the sequence relationship analyses, two other proteins might belong to the same cluster as the euANT/PLT proteins in rice, namely, AP2/EREBP22 and AP2/EREBP86 (Figure 1A and Supplementary Figure 2). The AP2/EREBP22 protein is somewhat divergent in its amino acid sequence compared to the other members of the *euANT/PLT* group and does not have a close relative in *A. thaliana*. In agreement with Li and Xue (2011), two clades within the euANT/PLT protein group were defined. Clade A contains OsPLT1∼6 proteins, which are more related to the *A. thaliana* PLT4/BABYBOOM (PLT4/BBM), PLT1, PLT2, and PLT5/AIL5 proteins plus AP2/EREBP86, which shows a close relationship with *A. thaliana* PLT3/AIL6 and PLT7/AIL7. Clade B contains the OsPLT7∼10 proteins that we observed to cluster with the AINTEGUMENTA (ANT) and AIL1 proteins, a close relationship being seen between ANT and the OsPLT7∼8 paralogs.

Our previously described RNAseq datasets reported numerous AP2 subfamily genes in rice that were expressed in the different panicle meristem types and/or at branching stages (Harrop et al., 2016, 2019). Transcripts from eight *euANT/PLT* genes were identified (Figure 1A and Supplementary Figure 3). These genes could be categorized into three groups according to their expression levels in panicle meristems: barely expressed (*OsPLT1*, *OsPLT2*, and *OsPLT10*), mildly expressed (*OsPLT7* and *AP2/EREBP86*); and highly expressed (*OsPLT8*, *OsPLT9*, and *AP2/EREBP22*) (Supplementary Figure 3). Moreover, the highly expressed genes exhibited differential expression between the different meristem types in the rice panicle (Supplementary Figure 3). The *OsPLT8* gene was more highly expressed in the RM compared to the other meristem types. In contrast, *OsPLT9* and *AP2/EREBP22* genes were characterized by higher-level expression in other meristem types (PBMs; elongated PB with AMs; SpMs) (Supplementary Figure 3). The mildly expressed genes *OsPLT7* and *AP2/EREBP86* displayed expression levels that were stable between the different panicle meristem types. The four remaining *euANT/PLT* genes, namely, *OsPLT3*∼*6*, were not detected in these transcriptomes.

For further analysis, we focused on the three *ANT*-related genes, namely, *OsPLT7*, *OsPLT8*, and *OsPLT9*, as well as *AP2/EREBP22*, which was differentially expressed between different panicle meristem types. A developmental time course analysis during early panicle development (i.e., stages preceding floral organ differentiation) was performed by using a single panicle per stage with three biological replicates obtained per data point (Figure 1B). Two phases of development can be broadly defined: the indeterminate phase (panicle with inflorescence and branch meristems) and the determinate phase (panicle with spikelet and floret meristems). *OsPLT8* was found to display higher expression during the indeterminate phase, whereas *OsPLT7*, *OsPLT9*, and *AP2/EREBP22* expression was higher during the determinate phase. However, the amplitude of differential expression is still quite low, especially for *OsPLT7*. Moreover, as reported in the study by Li and Xue (2011) and in publicly available databases, the rice *ANT-*related genes have different expression profiles in different tissues or organs (Supplementary Figure 4). There was a low tissue or organ specificity for *AP2/EREBP22*, and there was a higher expression of *OsPLT9* in the panicle. *OsPLT8* was highly expressed in the endosperm but at lower levels in the panicle and leaf. In contrast, *OsPLT7* was highly expressed in callus and at lower levels in the meristem and spikelet.

**FIGURE 2:**
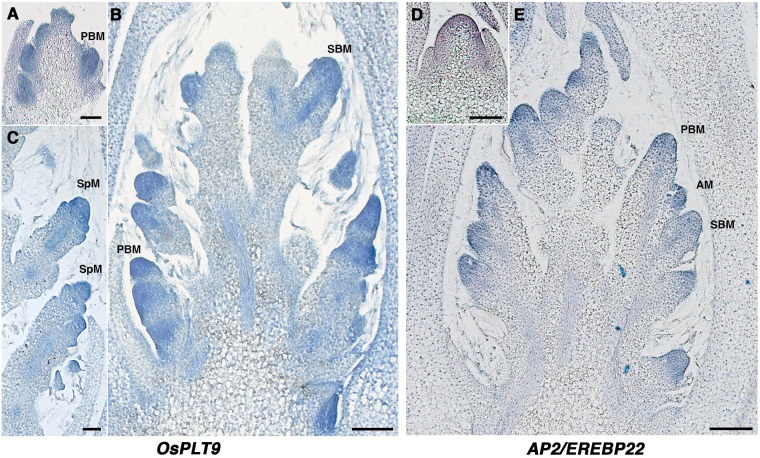
Spatial expression pattern of the *OsPLT9* and *AP2/EREBP22* genes in the young developing panicle using *in situ* hybridization. **(A)** *OsPLT9* transcript signal in the PBM. **(B)** *OsPLT9* transcript signal in the developing panicle with elongating primary branches bearing axillary meristems and secondary branches. **(C)** *OsPLT9* transcript signal in spikelet meristems. **(D)** *AP2/EREBP22* transcript signal in the rachis meristem. **(E)** *AP2/EREBP22* transcript signal in the developing panicle with elongating primary branches bearing axillary meristems and secondary branches. PBM, primary branch meristem; AM, axillary meristem; SBM, secondary branch meristem; SpM, spikelet meristem. Scale: 100 μm.

Using *in situ* hybridization analysis, the presence of transcripts of *OsPLT9* and *AP2/EREBP22* was confirmed during early development of the panicle of *O. sativa.* A signal was observed in both PBs and SBs and SpMs, throughout these meristems (Figure 2). *AP2/EREBP22* transcripts were also detected in the RM and *OsPLT9* transcripts in the vascular tissue of elongating branches (Figure 2). Unfortunately, it was not possible to obtain robust *in situ* hybridization signals for the *OsPLT7* and *OsPLT8* genes.

Overall, these data show that most of the *euANT/PLT* genes in *O. sativa* are expressed during early panicle development but with different expression patterns in terms of accumulation levels in panicle meristem types, notably for the paralogous *ANT*-like genes. This finding would suggest different functions for these genes during the panicle development.

### Opposing Effects of *OsPLT8/CRL5* Gene on PB and SB Formation

**FIGURE 3:**
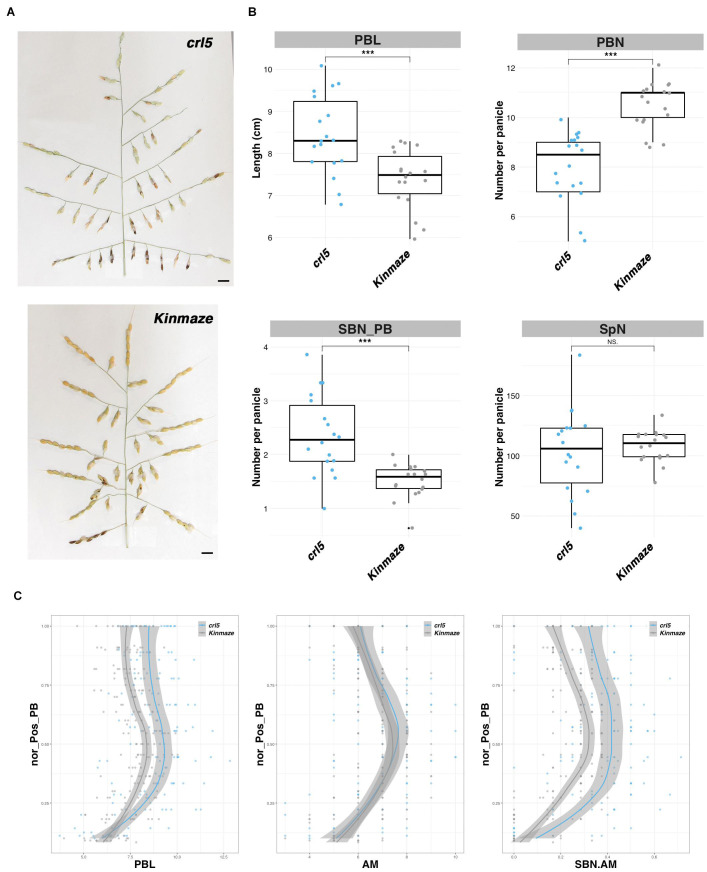
Panicle phenotype of the *crl5/plt8* mutant. **(A)** Mature panicles from the *crl5* mutant and its wild-type background (Kinmaze). Scale bar: 1 cm. **(B)** Box plots with individual dots indicating the average primary branch length (PBL), the number of primary branches (PBN), the ratio between secondary branch number and PBN (SBN_PB), and the spikelet number (SpN) per panicle of the *crl5* mutant, in comparison with wild-type plants of the same genetic background (Kinmaze). Individual dots in box plots correspond to average values from the three main panicles per plant. Statistical significance (*t*-test *p*-values) between the two lines or parents for the two panicle morphological traits is indicated as follows: NS if the test is non-significant; ***p-values < 0.001. **(C)** Plots of normalized positions of primary branches (Nor_pos_PB) against PBL, axillary meristem number per PB (AM), and the ratio of SBN vs. AM per PB (SBN.AM).

A phenotypic analysis of the loss-of-function *crl5* mutant panicle has been reported (Harrop et al., 2019) and was initially characterized on the basis of a defect in CR initiation (Kitomi et al., 2011). Here, a more detailed analysis is reported. The *crl5* mutant was found to be characterized by smaller plants and by a higher number of tillers and panicles per plant (Supplementary Figure 5). The *crl5* panicle, compared to that of the wild type (Figure 3A), showed a significant reduction in the total number of PBs plus longer PBs (Figure 3B). The total number of SBs was conserved. However, alongside the decrease in PB number, the number of SB per PB was increased in the *crl5* mutant compared to wild-type plants (Figure 3B). Overall, the total number of spikelets per panicle is not significantly affected in the mutant. A deeper analysis of the topology of the panicle, taking into account the relative positions of the PBs along the rachis, revealed that the length of the PBs is affected throughout the panicle (Figure 3C). The number of AMs per PB is not affected in the *crl5* mutant compared to wild type, even with longer PBs in *crl5* mutant (Figure 3C). However, the ratio of SB to AM numbers per PB is higher in the mutant (Figure 3C), indicating that the overall higher number of SB per PB (Figure 3B) results from a modification of the rate of AM determinate fate acquisition on the PBs. This modification influences the establishment of indeterminate SBMs in preference to determinate lateral SpMs on PBs.

Overall, the *OsPLT8/CRL5* gene appears to act as a positive regulator of PBM number and as a negative regulator of PB length and SBM activity.

### The *OsPLT9* Gene Acts as a Negative Regulator of AM Establishment and SB Formation on PB

**FIGURE 4:**
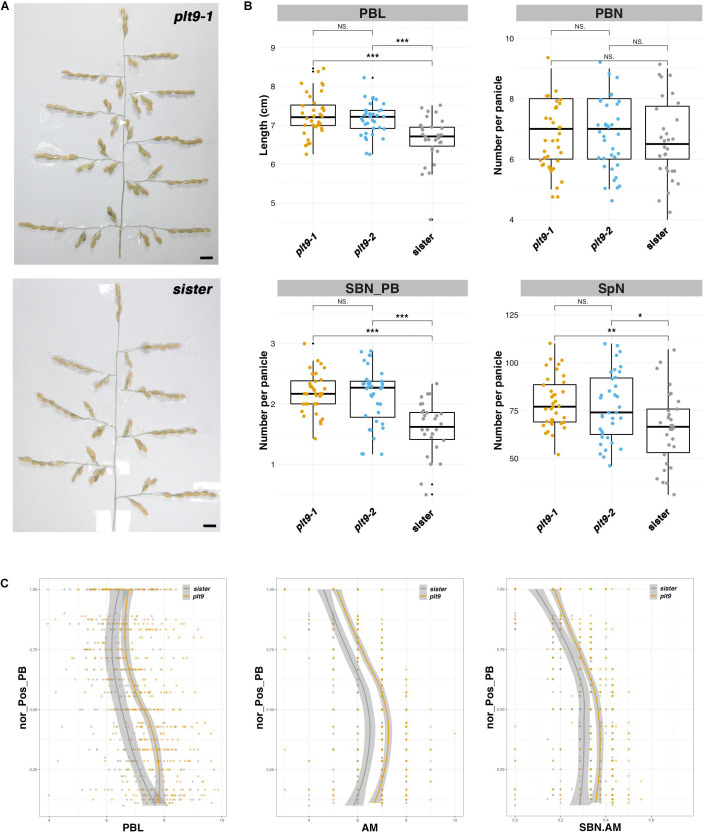
Panicle phenotype of the *plt9* mutant. **(A)** Mature panicles from the CRISPR-Cas9-derived *plt9-1* line and a sister (i.e., transgenic plant without mutation). Scale bar: 1 cm. **(B)** Box plots with individual dots indicating the average primary branch length (PBL), the number of primary branches (PBN), the ratio between secondary branch number and PBN (SBN_PB), and the spikelet number (SpN) per panicle of the CRISPR-Cas9-derived lines, in comparison with sister plants. Individual dots in box plots correspond to average values from the three main panicles per plant. Statistical significance (*t*-test *p*-values) between the two lines or parents for the two panicle morphological traits is indicated as follows: NS if the test is non-significant; *p-values < 0.05; **<0.01; ***<0.001. **(C)** Plots of normalized position of primary branch (Nor_pos_PB) against PBL, axillary meristem number per PB (AM), and the ratio of SBN vs. AM per PB (SBN.AM). The values of the *plt9-1* and *plt9-2* lines were pooled together as *plt9*.

Two CRISPR-Cas9-derived lines corresponding to different alleles of the *OsPLT9* gene were obtained, namely, *plt9-1* and *plt9-2*, as well as a sister line without a mutation (Supplementary Figure 6). The *plt9-1* allele corresponds to two deletions of 34 bp and 4 bp, respectively, leading to a truncated form of the protein lacking part of the second AP2 domain and the C-terminal region (Supplementary Figure 6). The *plt9-2* allele is characterized by a larger deletion of 84 bp, resulting in a deletion of 28 amino acids at the end of the second AP2 domain and the beginning of the C-terminal region of the protein (Supplementary Figure 6). The overall phenotypes of *plt9-1, plt9-2*, and the sister line plants were much alike with a similar height, numbers of tillers, and panicles (Supplementary Figure 5). The panicle phenotypes of *plt9-1* and *plt9-2* plants were similar in terms of PB length, and numbers of PBs, SBs, and spikelets (Figure 4). In comparison with the sister line, the two *plt9* lines were characterized by longer PBs (Figures 4B,C) and higher numbers of SBs per PB and total spikelets, but not affected in the number of PBs (Figure 4B). Similar to the *crl5* mutant, the ratio between SB and AM numbers per PB was higher in the two *plt9* lines, but in contrast to *crl5*, the longer PB of the *plt9* mutants was associated with an increase in AM number per PB (Figure 4C). Consequently, the higher numbers of SBs per PB could be related to the additive effects of a higher number of AMs per PB and an alteration of the transition rate from indeterminate to determinate fate in AMs on the PBs, as in *crl5* mutant.

Overall, the *OsPLT9* gene appears to act as a negative regulator of AM establishment on PBs and also of fate transition on SBMs, without any impact on AM establishment on rachis.

### The *AP2/EREBP22* Gene Acts as a Negative Regulator of AM Establishment Leading to Both PBs and SBs

**FIGURE 5:**
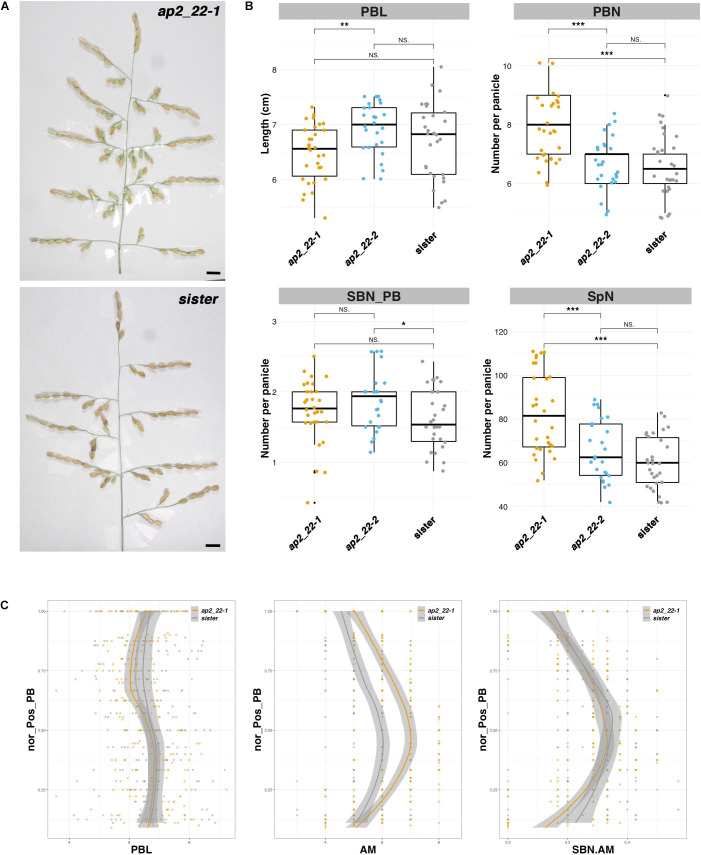
Panicle phenotype of the *ap2/erebp22* mutant. **(A)** Mature panicles from the CRISPR-Cas9-derived *ap2_22-1* line and a sister (i.e., transgenic plant without mutation). Scale bar: 1 cm. **(B)** Box plots with individual dots indicating the average primary branch length (PBL), the number of primary branches (PBN), the ratio between secondary branch number and PBN (SBN_PB), and the spikelet number (SpN) per panicle of the CRISPR-Cas9-derived lines, in comparison with sister plants. Individual dots in box plots correspond to average values from the three main panicles per plant. Statistical significance (*t*-test *p*-values) between the two lines or parents for the two panicle morphological traits is indicated as follows: NS if the test is non-significant; *p-values < 0.05; **<0.01; ***<0.001. **(C)** Plots of normalized position of primary branch (Nor_pos_PB) against PBL, axillary meristem number per PB (AM), and the ratio of SBN vs. AM per PB (SBN.AM). Only the values for the ap2_22-1 line were considered as being significantly different from the sister plants.

Two CRISPR-Cas9-derived lines corresponding to different alleles of the *AP2/EREBP22* gene were obtained, namely, *ap2_22-1* and *ap2_22-2*, as well as a sister line (Supplementary Figure 6). The *ap2_22-1* allele is characterized by two deletions of 1 and 3 bp, respectively, leading to a truncated form of the protein lacking part of the second AP2 domain and the C-terminal region (Supplementary Figure 6). The *ap2_22-2* allele is characterized by two deletions of 3 and 6 bp, respectively, resulting in an amino acid substitution and two single amino acid deletions within the second AP2 domain of the protein (Supplementary Figure 6). While *ap2_22-1* and sister line plants were similar in terms of height, tiller number, and panicle number, the *ap2_22-2* plants produced significantly more tillers and panicles than the sister line with a similar plant height (Supplementary Figure 5). In contrast, the panicles from *ap2_22-1* plants were distinct from the *ap2_22-2* and sister line plants (Figure 5). The total number of PBs and spikelets per panicle was higher in *ap2_22-1* plants. However, PB length and the number of SBs per PB were not altered. The number of AMs per PB was increased but not the ratio between SB and AM numbers per PB. It can therefore be concluded that the *AP2/EREBP22* gene acts upon AM establishment on both the rachis and PB but not on SB meristem fate control. Finally, both the *ap2_22-1* and *ap2_22-2* mutant lines showed an earlier flowering time of about 1 week compared to the sister line, suggesting a role for the *AP2/EREBP22* gene in the regulation of this process (Supplementary Table 3).

## Discussion

In rice, little is known about the role of *euANT*/*PLT* genes in meristem functioning. Recently, triple mutants were generated for the *OsPLT6, OsPLT5, and OsPLT3* genes (also known as *BBM1*∼*3*), allowing a demonstration that male genome-derived expression of *BBM1-OsPLT6* triggers the embryonic program in the fertilized egg cell and that this gene acts redundantly with other *BBM* genes (Khanday et al., 2019). Additionally, *OsPLT5*, in combination with the *WUS2* gene, promotes regeneration from tissue culture, suggesting that they function as pluripotency factors in grass species, including *O. sativa* (Lowe et al., 2016). Expression profiling in rice roots of *OsPLT1*∼*6* revealed that these genes are up-regulated both in the primordia of CRs and in developing CRs (Garg et al., 2020; Lavarenne et al., 2020). Moreover, these *euANT/PLT* clade A genes are specifically expressed in different sectors of the CR meristem (Li and Xue, 2011; Kitomi et al., 2018). This suggests that these transcription factors might be the important regulators in different phases of CR primordium development. Recently, *OsPLT1* and *OsPLT4* were shown to play a role in CR outgrowth (Garg et al., 2020). In contrast, *euANT/PLT* clade B genes including *OsPLT8/CRL5* might play a key role during CR initiation without being involved in the later steps of CR primordia differentiation (Garg et al., 2020; Lavarenne et al., 2020). In this sense, *OsPLT8/CRL5* was initially demonstrated to be involved in the auxin-dependent *de novo* pathway of CR initiation in rice, through the repression of cytokinin signaling by positively regulating the type-A RR OsRR1 (Kitomi et al., 2011).

**FIGURE 6:**
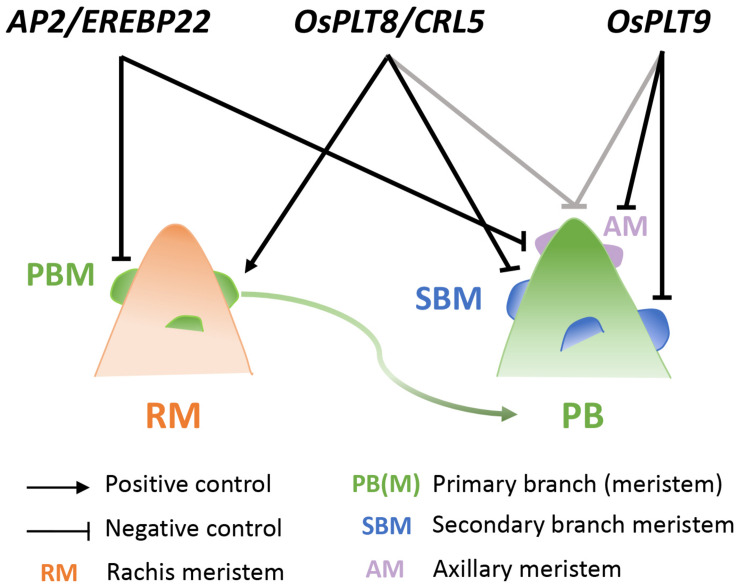
Model of *OsPLT8, OsPLT9, and AP2/ERBP22* gene interactions in panicle branching complexity in relation to meristem fate control. Schematic view of the mode of action of the *OsPLT8, OsPLT9*, and *AP2/ERBP22* genes on the rachis (left panel) and the primary branch (right panel). Black arrow indicates a positive action on axillary meristem establishment on rachis. Blunt ends in black indicate the negative actions on axillary meristem establishment and/or axillary meristem fate transition. Blunt ends in gray indicate the negative action on the primary branch length. AM, axillary meristem (on PB); PB, primary branch; PBM, primary branch meristem; RM, rachis meristem; SBM, secondary branch meristem.

Most of the aforementioned genes are also expressed in young developing panicles (Harrop et al., 2016, 2019). This suggests an overlap between the regulatory mechanisms in root and panicle systems for the establishment and functioning of *de novo* meristems leading to branching in these plant structures. Our present study demonstrated a role for *OsPLT8/CRL5*, *OsPLT9*, and *AP2/EREBP22* in the control of panicle architecture. These closely related paralogous genes exert shared and sometimes divergent influences on meristem establishment and fate, indicating a partial functional diversification (Figure 6). Although no distinct spatial expression pattern within the meristem was evidenced for *OsPLT9* and *AP2/EREBP22*, the *ANT*-related genes as a whole display temporally differential expression profiles during the early panicle development. The *OsPLT8/CRL5* gene was expressed at a higher level during indeterminate stages, in contrast to *OsPLT7*, *OsPLT9*, and *AP2/EREBP22* with higher level expression during the determinate stages. Moreover, the *OsPLT8/CRL5* gene acts as both a positive regulator of PBMs and a negative regulator of SBMs. Indeed, this gene promotes AM establishment on the rachis but not on PBs. Furthermore, it has a role in promoting AM fate transition from indeterminate to determinate fate on PBs, leading to the establishment of spikelets rather than of SBs. In contrast, the *OsPLT9* gene has no effect on AM establishment leading to PBs on the rachis, but acts as a negative regulator of AM initiation and SB formation on PBs. Similar to *OsPLT8/CRL5*, *OsPLT9* might promote the transition from indeterminate to determinate fate in the AMs of PBs, suggesting a partial overlap in the function of these two genes in this process. In addition, the *OsPLT8/CRL5* and *OsPLT9* genes appear to have an overlapping negative influence on the PB length. In contrast to the other two genes, *AP2/EREBP22* acts as a negative regulator of AM establishment on both the rachis and PB without any impact on the transition from indeterminate to determinate fate in AMs, nor on PB length. Consequently, it can be concluded that *OsPLT8/CRL5* and *AP2/EREBP22* have antagonist influences upon AM establishment on the rachis.

For the *OsPLT9* gene, the *plt9-1* mutant allelic form encoded a truncated protein lacking the C-terminal domain with the second AP2 domain partially modified, whereas the protein encoded by the *plt9-2* mutant is characterized by an alteration of the C-end of the second AP2 domain but a largely conserved C-terminal region. The similar phenotypes observed between the *plt9-1* and *plt9-2* mutant plants indicate that the integrity of the second AP2 domain is more important than that of the C-terminal domain for the biological activity of OsPLT9 during the panicle development. For *AP2/EREBP22*, the *ap2_22-1* mutant allele encoded a truncated form of the protein lacking the second AP2 domain and the short C-terminal region, whereas the *ap2_22-2* allele specified a protein carrying only a three-amino acid deletion plus a substitution in the second AP2 domain. Similar to OsPLT9, the integrity of the second AP2 domain is important for the biological activity of AP2/EREBP22 during panicle development. The significant increase in tiller and panicle production without altering the panicle morphological traits in the *ap2_22-2* line would suggest that this domain might still be functional at least for its binding activity to specific target genes involved in the panicle development. However, it might also be affected in its affinities for DNA-binding or protein partners targeting specific genes related to tillering, which in turn affects the number of panicles produced.

Collectively, our results indicate that the rice *ANT* homologs *OsPLT8/CRL5* and *OsPLT9*, along with *AP2/EREBP22*, display partial functional diversification, with certain opposing effects, in governing the branching of the panicle. The *OsPLT9*, *AP2/EREBP22*, *OsPLT8/CRL5*, and *OsPLT7* genes are all expressed within the panicle meristems as witnessed by *in situ* hybridization (present study) and by meristem-specific RNAseq data (Harrop et al., 2016). This suggests that their partial functional diversification might be determined more by the differential affinity of the transcription factors to specific target genes and/or protein–protein interactions within larger regulatory complexes than to their spatial or temporal patterns of accumulation. Furthermore, in addition to the role of *OsPLT8/CRL5* in the CR initiation, the genes described in this study might have roles in other developmental processes. Mutant lines for *AP2/EREBP22* have been shown to display an altered flowering time, suggesting an additional role for this gene in the control of the transition from the vegetative to the reproductive phase. Moreover, OsPLT8/CRL5 and AP2/EREBP22 appear to also play a role in vegetative meristems, as illustrated by the alterations in tiller number displayed by their respective mutants. Unfortunately, we were unable to obtain plants carrying CRISPR-Cas9-induced mutations for the *OsPLT7* gene after transformation. It cannot be excluded that the designed gRNA sequences were not functional. However, our observations could also be explained by the fact that this gene is highly expressed in callus (see Supplementary Figure 4). The loss of function of this gene might therefore impact upon callogenetic capability during the *in vitro* culture process used for rice genetic transformation, leading to the selection of non-mutated transgenic plants.

There are still several outstanding questions concerning the roles of the presently described *PLT* genes in panicle development that remain to be addressed. It will clearly be of interest to identify the target genes of these transcription factors and to study their interactions with other molecular factors affecting the panicle architecture through AM establishment and/or meristem fate transition (e.g., hormones, other genes). In *A. thaliana*, prolonged high auxin levels were found to be required to promote PLT activities (Mähönen et al., 2014). Moreover, *OsPLT8/CRL5* was shown to be induced by auxin and to act through the repression of cytokinin signaling (Kitomi et al., 2011), suggesting that a similar regulatory pathway might operate in panicle meristems. Cytokinins were also shown to play a role in rice panicle branching, as reported by the characterization of the *Gn1a/OsCKX2, LONELY GUY (LOG)* genes involved in cytokinin signaling (Ashikari et al., 2005; Kurakawa et al., 2007), as well as other cytokinin-related genes (Han et al., 2014; Yamburenko et al., 2017). The exact role of auxin and its mode of action during the early stages of panicle development in rice remain to be elucidated. However, auxin maxima and dynamic re-localization have been observed at the initiation sites of inflorescence and spikelet primordia, including branch meristems and female and male organs (Yang et al., 2017). In this context, it will be of importance to establish the link between the *euANT/PLT* genes and the two aforementioned hormones during the early panicle development. Transcriptomic analyses of the different meristem types in rice panicle and between different species from Oryza genus have allowed the identification of co-expressed genes that could be good candidates for further analysis (Harrop et al., 2016, 2019). Moreover, the same studies revealed that other members of the *euANT/PLT* clade are expressed during the panicle development. It will be of interest to identify their roles during panicle development and their genetic interactions, so as to evaluate their importance in determining the panicle architecture diversity within the genus *Oryza* and in crop and wild species as a whole.

## Data Availability Statement

The original contributions presented in the study are included in the article/Supplementary Material, further inquiries can be directed to the corresponding author/s.

## Author Contributions

HA and SJ contributed to the conception and design of the experiments. JT supervised the initial project. AL, HA, and CG performed the expression analysis experiments. AL, NK, QL, TL, and ML performed CRISPR-Cas9 constructs in Vietnam. AL, HA, PA, and MF participated in transgenic plant production and management in France. AL, FN, and SJ performed the phenotypic analysis of the plants. AL and SJ analyzed the data and wrote the manuscript. HA and JT revised the manuscript. All authors have read and approved the final manuscript.

## Conflict of Interest

The authors declare that the research was conducted in the absence of any commercial or financial relationships that could be construed as a potential conflict of interest.

## Footnotes

1 https://www.ncbi.nlm.nih.gov/gene/

2 www.evolgenius.info
