# Supplementary material for: Functional Diversification of euANT/PLT Genes in Oryza sativa Panicle Architecture Determination
Source: Front Plant Sci. 2021 Jul 9;12:692955. doi: 10.3389/fpls.2021.692955 (PMC8302143; doi:10.3389/fpls.2021.692955)
Supplement: Supplementary file 1 [file Data_Sheet_1.PDF]

# *Supplementary Material*

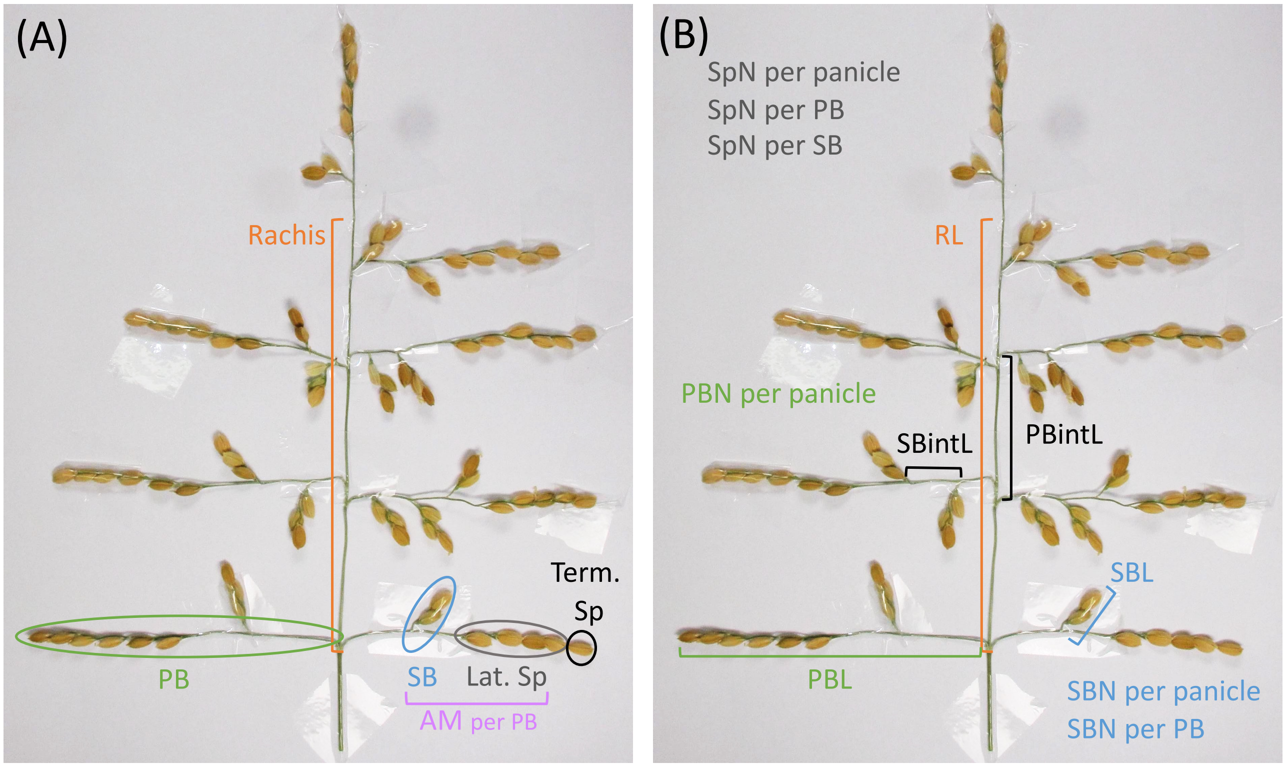


**Supplementary Figure 1. Morphological trait components of the panicle and measured traits using P-TRAP software.** Spread mature panicle from *O. sativa* cv. Kitaake with morphological trait components **(A)** and the measured traits using P-TRAP software **(B)**. AM: axillary meristem; PB: primary branch; PBL: average length of PB per panicle; PBN: PB number per panicle; PBintL: average length of internodes on rachis per panicle; RL: rachis length; SB: secondary branch; SBL: average length of SB per panicle; SBN: SB number per panicle and per PB; SBintL: average length of internodes on PB per panicle; SpN: spikelet number per panicle or per PB or per SB; Lat. Sp: lateral spikelets of PB; Term. Sp: terminal spikelet of PB.

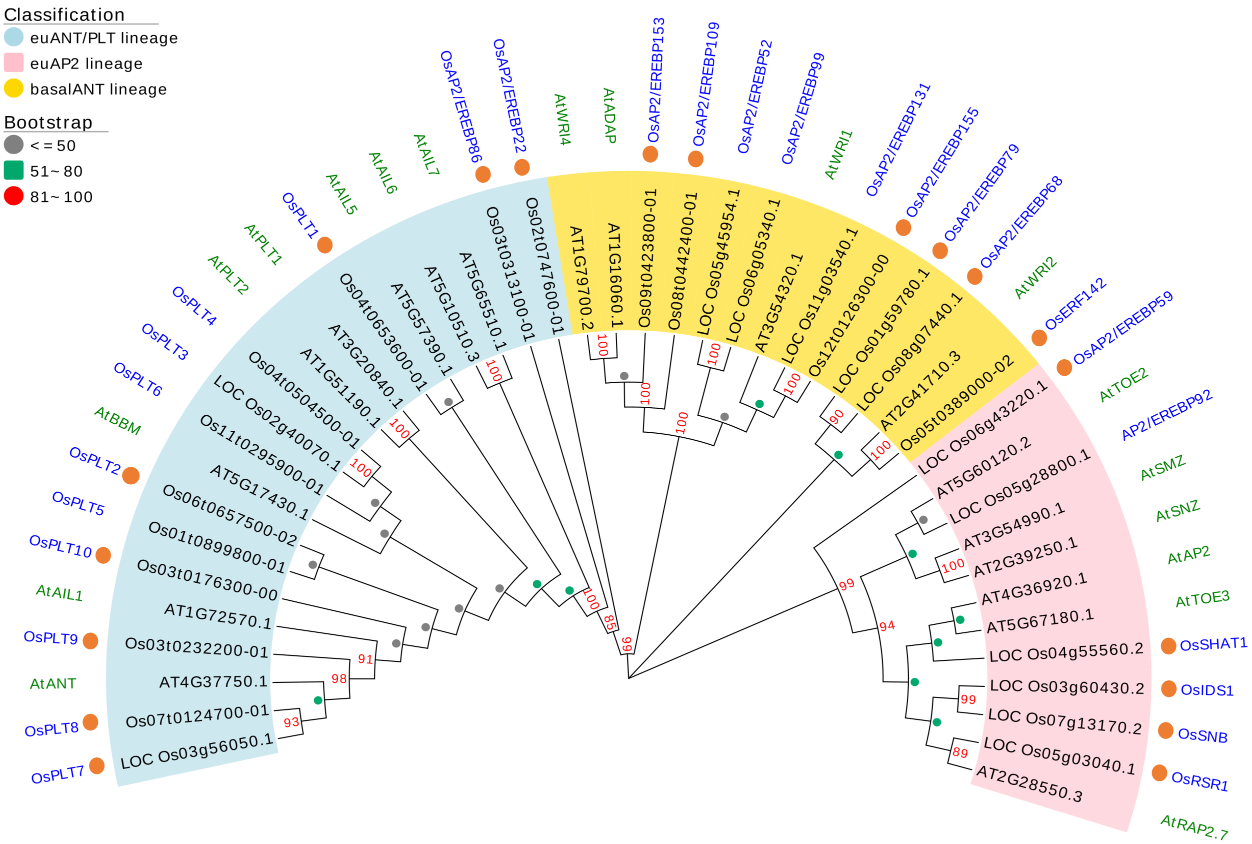


**Supplementary Figure 2. An unrooted phylogenetic tree of the AP2 subfamily in *Oryza sativa ssp. japonica*.** 45 amino acid sequences of the AP2 subfamily were aligned using MEGA7, and the phylogenetic tree was constructed using the NJ method with the following default parameters: bootstrap method (1000 replicates), p-distance correction, uniform rates and pairwise deletion. The blue, yellow and pink background indicated the euANT/PLT, basalANT and euAP2 group, respectively. Orange circles indicate those genes expressed in panicle at early stage and detected in two RNA-seq datasets.

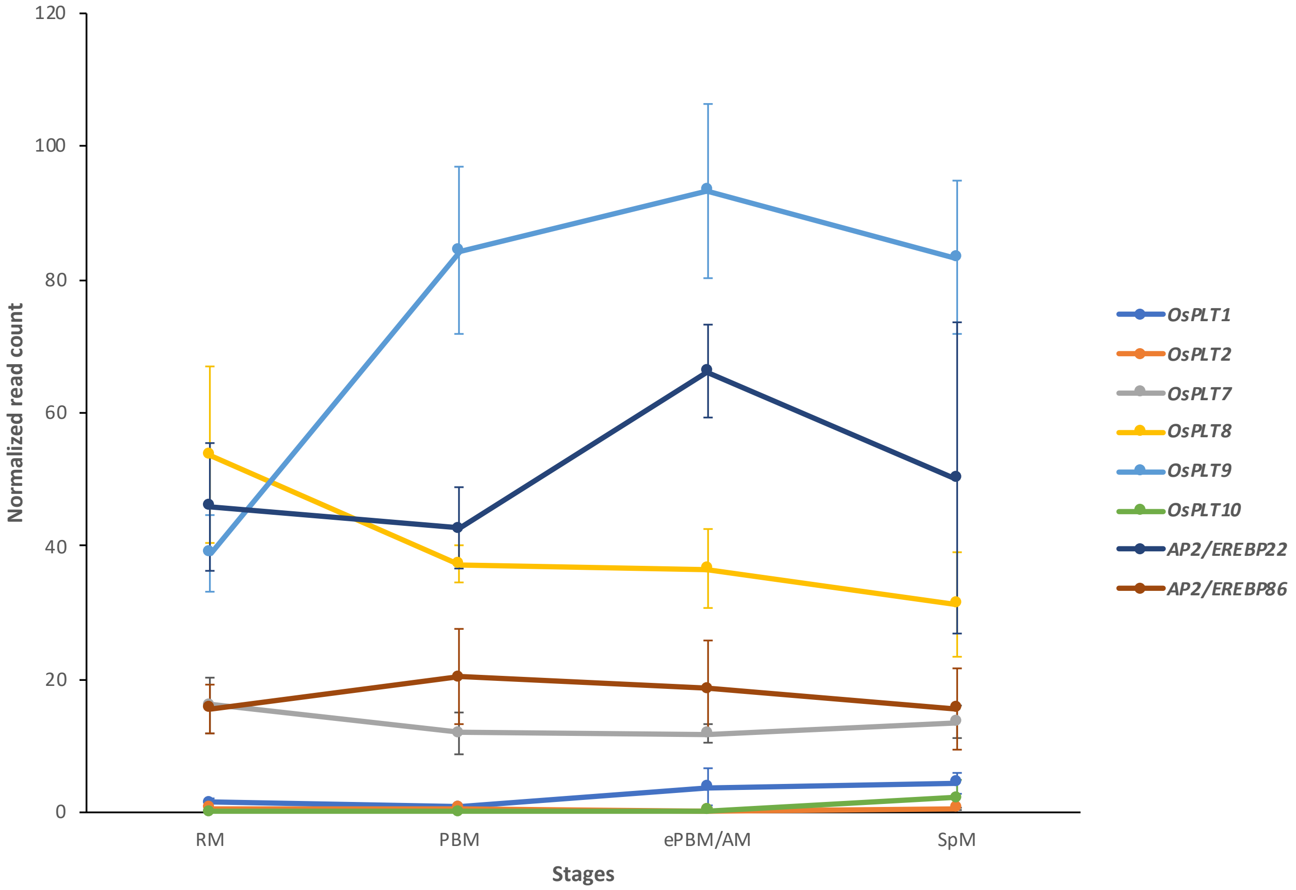


**Supplementary Figure 3. Expression profiling of the *euANT/PLT* genes in panicle meristems of *O. sativa* cv. Nipponbare.** Normalized read count from RNAseq dataset on laser-dissected panicle meristems: RM, rachis meristem; PBM, primary branch meristem; ePBM/AM, elongated primary branch meristem with axillary meristems; SpM, spikelet meristem. Data from Harrop et al. (2016).

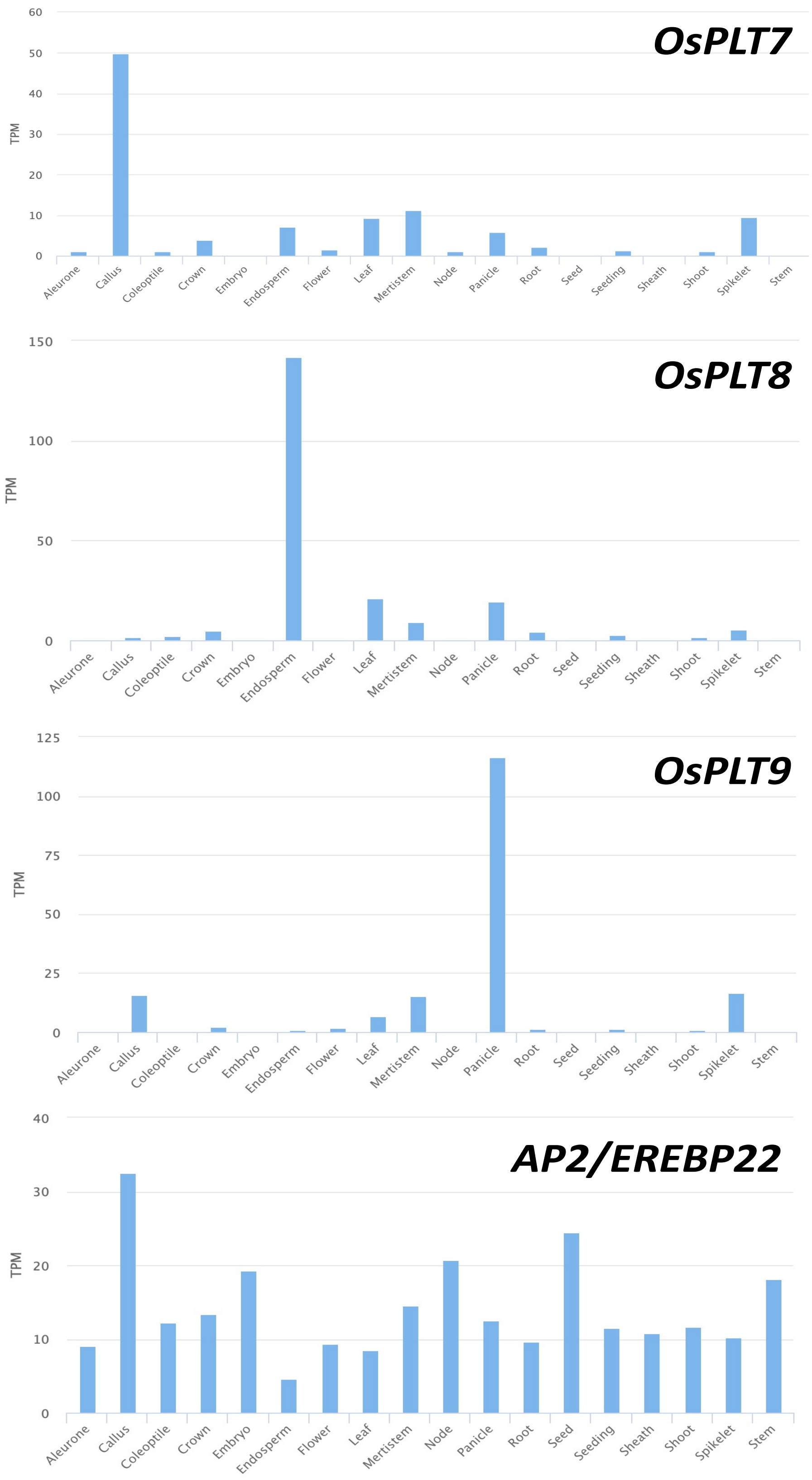


**Supplementary Figure 4. Expression profiling of the *OsPLT7*, *OsPLT8*, *OsPLT9* and *AP2/EREBP22* genes in *O. sativa* cv. Nipponbare.** Expression profiles in various tissues or organs according to data available in IC4R website (http://www.ic4r.org). TPM: transcripts per million.

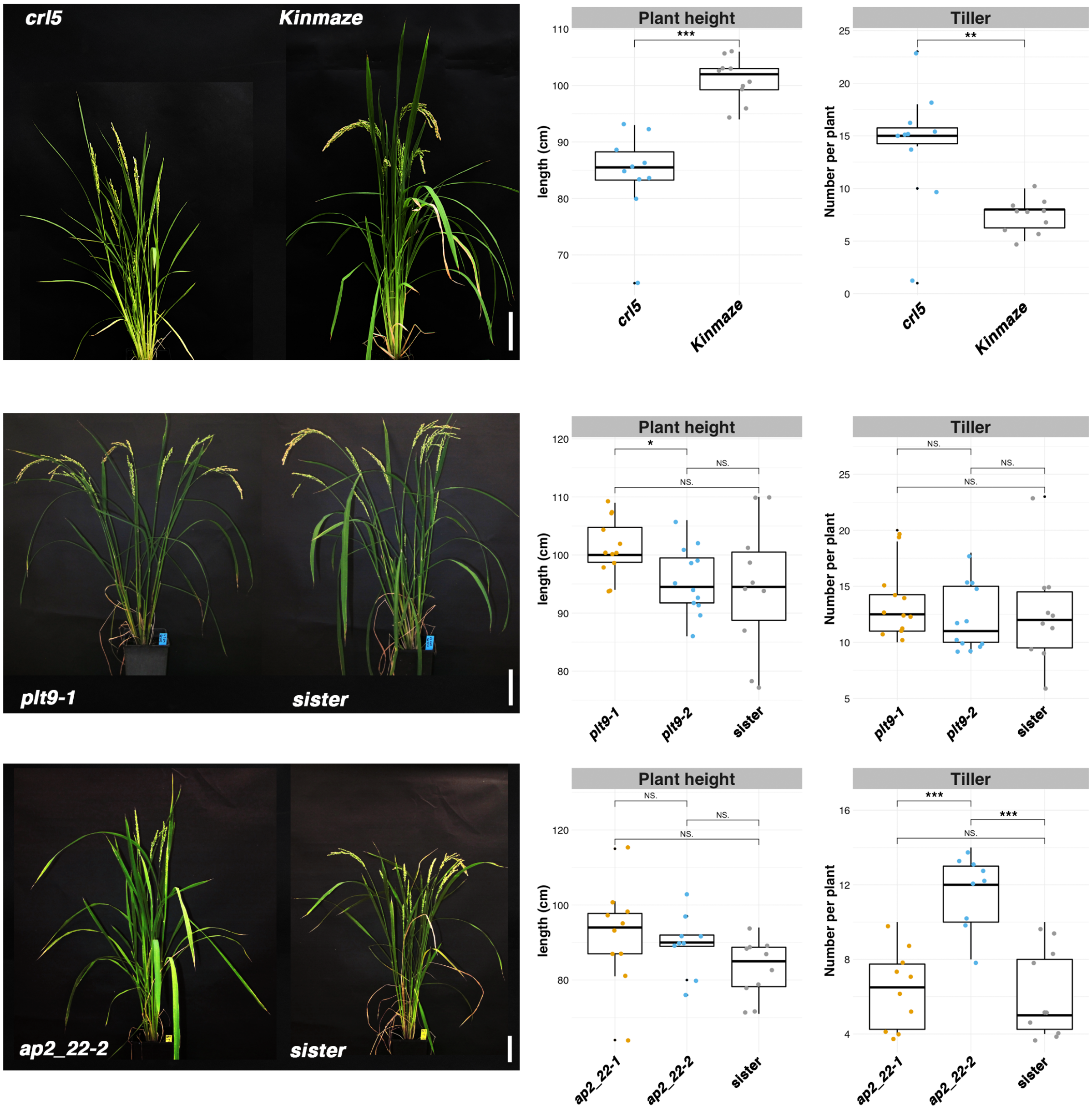
crl5
Kinmaze
Plant height
length (cm)
Tiller
Number per plant
plt9-1
sister
plt9-2
NS.
ap2_22-2
ap2_22-1

**Supplementary Figure 5. Phenotype at vegetative stage of the *plt8*/*crl5*, *plt9* and *ap2/erebp22* mutants.** Whole plant morphology of the mutants and corresponding wild-type or sister (i.e. transgenic plant without mutation) backgrounds are shown in the left panel. Plant height and tiller number per plant are indicated by box-plots on the right panel. Individual dots in box-plots correspond to average values from the 3 main panicles per plant. Statistical significance (*t*-test *p* values) between the two lines or parents for the two panicle morphological traits is indicated as follows: NS if the test is non-significant; * if *p*-values <0.05; ** if <0.01; *** if <0.001.

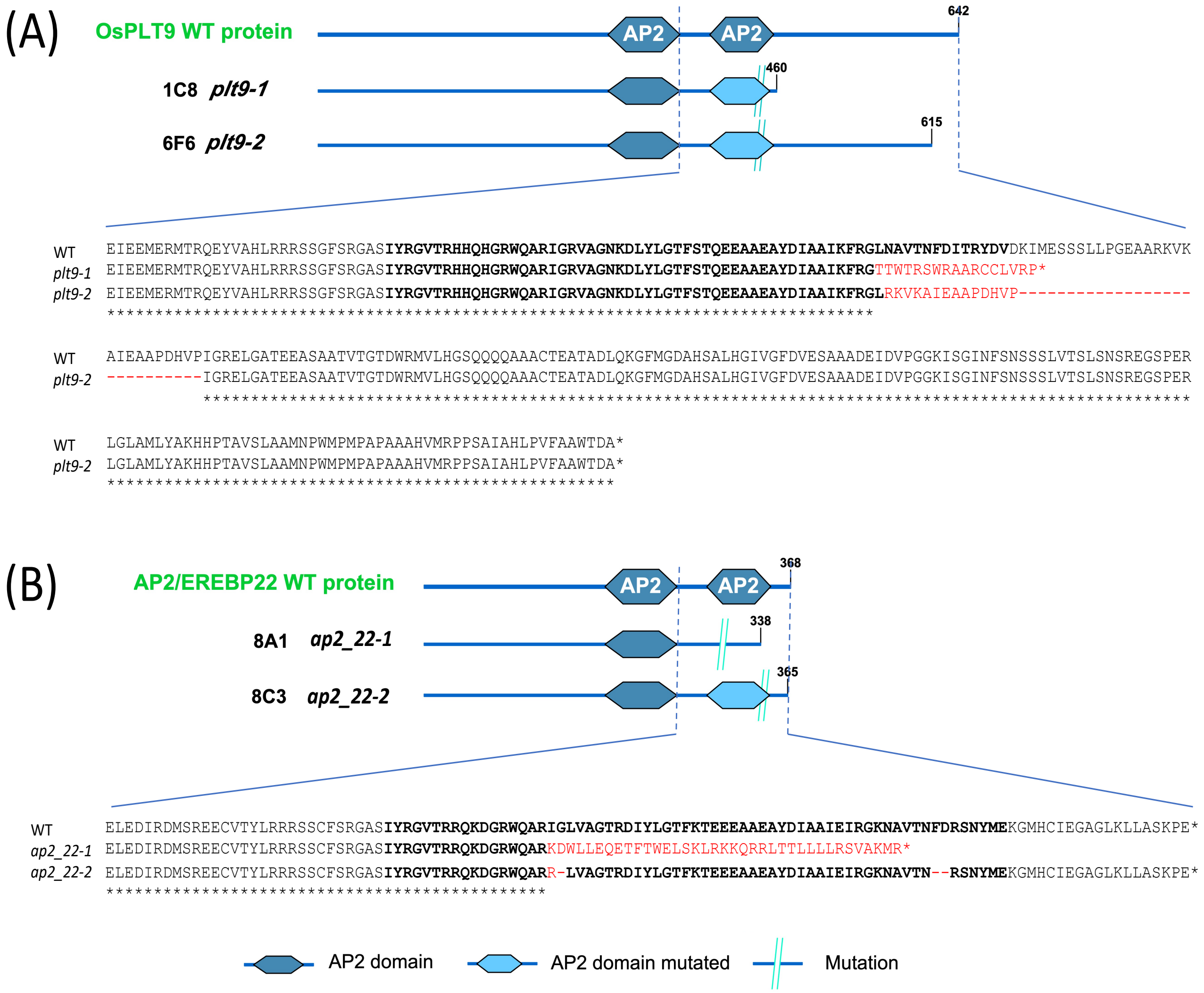


**Supplementary Figure 6. Predicted protein sequence changes in the edited mutants of *OsPLT9 and AP2/EREBP22* genes.** Schemas describing the protein structure with protein domain for each mutant line of *OsPLT9* (A) and *AP2/EREBP22* (B), respectively, along with representative sequences of the protein deletion aligned with that of wild-type (WT). The blue lines indicate the protein sequence with the length of protein was shown in the tail. The light blue strips represent the mutation positions. The dark blue box represents the normal AP2-binding domain. The light blue box represents the mutant AP2-binding domain. *OsPLT9* mutant lines: 1C8, *plt9-1*, 6F6, *plt9-2. OsAP2/EREBP22* mutant lines: 8A1, *ap22-1*, 8C3, *ap22-2.* The second AP2 domain sequence is indicated by bold letters in the amino acid sequence alignments. Red letters indicate the protein changes in the mutants compared to WT.

# Supplementary Table 1. List of the primers used in this study.

| Oligo Name | Sequence (5'-3') | Purpose |
|---|---|---|
| PLT9-Geno-F | ACGGAGCGTTTCCATTGGTT | Genotyping and sequencing of CRISPR Cas9 lines |
| PLT9-Geno-R | GTGCCCGTGACAGTAGCAG | |
| PLT9-Seq-F | TTTATGCAATACAGGCACTCAAG | |
| PLT9-Seq-R | CTCGCTTCCTCGGTCGC | |
| AP22-Geno-F | CTTCAGTTTGTTGCCAAGGCT | |
| AP22-Geno-R | CCCTAATATGCGATGCGGCT | |
| AP22-Seq-F | CTCTGCTAAACCATGCCCCT | |
| AP22-Seq-R | CTTCAAGCCTGCCCCTTCTAT | |
| Hygro-F | GCTCCAGTCAATGACCGCTG | Hygromycin phospho-transferase II detection |
| Hygro-R | CTCGGAGGGCGAAGAATCTC | |
| UBI-F | GCTTGTGCGTTTCGATTTGA | Detection of *Cas9* |
| Cas9-R | CCGCTCGTGCTTCTTATCCT | |
| PLT8a-F | TAGGTCTCCAGCGGTTTCTCAGTTTTAGAGCTAGAA | PTG synthesis CRISPR-Cas9 |
| PLT8a-R | ATGGTCTCACGCTACTTTTCCTGCACCAGCCGGGAA | |
| PLT8b-F | TAGGTCTCCCATCAGCACGGAGTTTTAGAGCTAGAA | |
| PLT8b-R | ATGGTCTCAGATGATGCCTGCTGCACCAGCCGGGAA | |
| PLT9a-F | TAGGTCTCCGTGGCCTGAACGGTTTTAGAGCTAGAA | |
| PLT9a-R | ATGGTCTCACCACGGAACTTGTGCACCAGCCGGGAA | |
| PLT9b-F | TAGGTCTCCAGGCAGCGCGTAGTTTTAGAGCTAGAA | |
| PLT9b-R | ATGGTCTCAGCCTCACCAGGCTGCACCAGCCGGGAA | |
| PLT7a-F | TAGGTCTCCGCGGCGGATCGTACTAGTTTTAGAGCTAGAA | |
| PLT7a-R | ATGGTCTCACCGCCGGCTGCACCAGCCGGGAA | |
| PLT7b-F | TAGGTCTCCACAGCGCACTGCGTTTTAGAGCTAGAA | |
| PLT7b-R | CGGGTCTCACTGTCATCCCCGTGCACCAGCCGGGAA | |
| AP2-22a-F | TAGGTCTCCCACGCATAGGACGTTTTAGAGCTAGAA | |
| AP2-22a-R | CGGGTCTCACGTGCCTGCCACTGCACCAGCCGGGAA | |
| AP2-22b-F | TAGGTCTCCTTCGATCAAAGTGTTTTAGAGCTAGAA | |
| AP2-22b-R | CGGGTCTCACGAAGCAACTACTGCACCAGCCGGGAA | |
| UGW-U3-F | GACCATGATTACGCCAAGCTTAAGGAATCTTTAAACATACG | |
| UGW-gRNA-R | GGACCTGCAGGCATGCACGCGCTAAAAACGGACTAGC | |
| L5AD5-F | CGGGTCTCAGGCAGGATGGGCAGTCTGGGCAACAAAGCACCAGTGG | |
| L3AD5-R | TAGGTCTCCAAACGGATGAGCGACAGCAAACAAAAAAAAAAGCACCGACTCG | |
| S5AD5-F | CGGGTCTCAGGCAGGATGGGCAGTCTGGGCA | |
| S3AD5-R | TAGGTCTCCAAACGGATGAGCGACAGCAAAC | |
| CRL5-insitu-F | ACGACGTGGCGGCGATCAAG | *In situ* hybridization probes |
| CRL5-insitu-R | TTAGGCGTCGGTCCAGGCGG | |
| CRL5-insitu-T7-F | GCGAAATTAATACGACTCACTATAGGGCGAAACGACGTGGCGGCGATCAAG | |
| CRL5-insitu-T7-R | GCGAAATTAATACGACTCACTATAGGGCGAATTAGGCGTCGGTCCAGGCGG | |
| AP22-new-pro-F | ACCACGAAGGGAGTTGAGTC | |
| AP22-new-pro-R | GCCGTAGTTCCAGCAGTACC | |
| AP22-new-pro-T7-F | GCGAAATTAATACGACTCACTATAGGGCGAAACCACGAAGGGAGTTGAGTC | |
| AP22-new-pro-T7-R | GCGAAATTAATACGACTCACTATAGGGCGAAGCCGTAGTTCCAGCAGTACC | |
| PLT9-Sens | TATAGCCAGGGCCAAGAAGC | |
| PLT9-ASens | GCTGGTGACGAGGAAGTTCT | |
| PLT9-pT7sens | GCGAAATTAATACGACTCACTATAGGGCGAATATAGCCAGGGCCAAGAAGC | |
| PLT9-pT7AS | GCGAAATTAATACGACTCACTATAGGGCGAAGCTGGTGACGAGGAAGTTCT | |
| T7-HIS | GCGAAATTAATACGACTCAC | |
| PLT7-F | TGGCTCACCTCAGAAGGAA | qRT-PCRs |
| PLT7-R | TCCTCCTGCGTGCTGAATGT | |
| PLT8-F | TAGGGTTCTTGGTTGCTCGG | |
| PLT8-R | CGGAGAAGAAGGAAAGGTGG | |
| PLT9-F | CGAGAGAGCAACGCAAGAAC | |
| PLT9-R | AGAGCGAGAAGCCTAACCAG | |
| EREBP22-F | CTCGGTAGTTGATTCCTCCC | |
| EREBP22-R | CACCAATCAATCGCTCTACCC | |

**Supplementary Table 2. Features of *O. sativa euANT/PLT* genes and proteins in RAPdb, MSU and NCBI databases.** The *ANT* gene homologues and AP2/EREBP22 gene are highlighted. *: NCBI protein IDs corresponding to the same isoform (i.e. same amino acid sequence). RAPdb: https://rapdb.dna.affrc.go.jp/index.html; MSU: http://rice.plantbiology.msu.edu; NCBI: https://www.ncbi.nlm.nih.gov/gene/.

| Gene symbol | RAPdb | | | | | MSU | | | | | NCBI | | | | | |
|---|---|---|---|---|---|---|---|---|---|---|---|---|---|---|---|---|
| | Gene locus | Gene length (bp) | Transcript ID | CDS length (bp) | Protein length (aa) | Gene locus | Gene length (bp) | Transcript ID | CDS length (bp) | Protein length (aa) | Gene locus | Gene length (bp) | Transcript ID | Transcript length (bp) | Protein ID | protein length |
| ***OsPLT1*** | Os04g0653600 | 4551 | Os04t0653600-01 | 1488 | 495 | LOC_Os04g55970 | 4585 | LOC_Os04g55970.1 | 828 | 275 | LOC4337245 | 4735 | XM_026025206.1 | 2357 | XP_025880991.1 | 502 |
| | | | | | | | | LOC_Os04g55970.2 | 1479 | 492 | | | XM_015778215.2 | 2327 | XP_015633701.1 | 495 |
| ***OsPLT2*** | Os06g0657500 | 3855 | Os06t0657500-01 | 573 | 190 | LOC_Os06g44750 | 3903 | LOC_Os06g44750.1 | 792 | 263 | LOC434172 | 5113 | XM_015786089.2 | 3161 | XP_015641575.1 | 469 |
| | | | Os06t0657500-02 | 1410 | 469 | | | | | | | | | | | |
| | | | Os06t0657500-03 | 792 | 263 | | | | | | | | | | | |
| ***OsPLT3*** | Os02g0614300 | 2094 | Os02t0614300-01 | 966 | 321 | LOC_Os02g40070 | 4627 | LOC_Os02g40070.1 | 2103 | 700 | LOC4329975 | 4772 | XM_015768488.2 | 2719 | XP_015623974.1 | 703 |
| ***OsPLT4*** | Os04g0504500 | 4264 | Os04t0504500-01 | 1977 | 658 | LOC_Os04g42570 | 4475 | LOC_Os04g42570.1 | 1977 | 658 | LOC9266143 | 4481 | XM_015780067.2 | 2584 | XP_015635553.1 | 658 |
| ***OsPLT5*** | Os01g0899800 | 4343 | Os01t0899800-01 | 2088 | 695 | LOC_Os01g67410 | 4350 | LOC_Os01g67410.1 | 2088 | 695 | LOC4325035 | 4547 | XM_015778958.2 | 2959 | XP_015634444.1 | 695 |
| ***OsPLT6*** | Os11g0295900 | 5214 | Os11t0295900-01 | 1680 | 559 | LOC_Os11g19060 | 5459 | LOC_Os11g19060.1 | 1680 | 559 | LOC4350315 | 5288 | XM_026021162.1 | 2211 | XP_025876947.1 | 499 |
| ***OsPLT7*** | Os03g0770700 | 3683 | Os03t0770700-01 | 1020 | 339 | LOC_Os03g56050 | 3581 | LOC_Os03g56050.1 | 1959 | 652 | LOC4334257 | 3963 | XM_026023764.1 | 2540 | XP_025879549.1 | 656 |
| | | | | | | | | | | | | | XM_026023765.1 | 2537 | XP_025879550.1 | 655 |
| ***OsPLT8*** | Os07g0124700 | 4702 | Os07t0124700-01 | 945 | 315 | LOC_Os07g03250 | 4702 | LOC_Os07g03250.1 | 945 | 314 | LOC4342308 | 6331 | XM_026026810.1 | 2844 | XP_025882595.1 | 643 |
| | | | Os07t0124700-02 | 1920 | 640 | | | | | | | | XM_026026812.1 | 2839 | XP_025882597.1 | 642 |
| ***OsPLT9*** | Os03g0232200 | 4303 | Os03t0232200-01 | 1929 | 642 | LOC_Os03g12950 | 4306 | LOC_Os03g12950.1 | 981 | 326 | LOC4332156 | 4705 | XM_015773923.2 | 2915 | XP_015629409.1 | 643 |
| | | | | | | | | | | | | | XM_015773924.2 | 2911 | XP_015629410.1 | 642 |
| ***OsPLT10*** | Os03g0176300 | 2330 | Os03t0176300-00 | 510 | 169 | LOC_Os03g07940 | 4689 | LOC_Os03g07940.1 | 1242 | 413 | LOC9268480 | 4789 | XM_015773904.2 | 2291 | XP_015629390.1 | 549 |
| ***AP2/EREBP22*** | Os02g0747600 | 3624 | Os02t0747600-01 | 1017 | 338 | LOC_Os02g51300 | 4273 | LOC_Os02g51300.1 | 741 | 246 | LOC4330724 | 4773 | XM_015770942.2 | 2142 | XP_015626428.1* | 367 |
| | | | | | | | | | | | | | XM_015770943.2 | 2386 | XP_015626429.1* | 367 |
| | | | | | | | | | | | | | XM_015770944.2 | 2403 | XP_015626430.1* | 367 |
| | | | | | | | | | | | | | XM_015770945.2 | 2438 | XP_015626431.1 | 366 |
| ***AP2/EREBP86*** | Os03g0313100 | 5168 | Os03t0313100-01 | 1296 | 431 | LOC_Os03g19900 | 5170 | LOC_Os03g19900.1 | 1473 | 490 | LOC4332640 | 5509 | XM_015773788.2 | 2005 | XP_015629274.1 | 431 |

**Supplementary Table 3. Summary of the flowering time of *crl5*, *plt9* and *ap2_22* mutants and wild-type relatives.** DAG: day after growing.

| Genotype | 1st flowering (DAG) | 50% flowering (DAG) | 100% flowering (DAG) |
|---|---|---|---|
| *crl5* | 78 | 83 | 96 |
| Kinmaze | 83 | 84 | 96 |
| *plt9-1* | 45 | 48 | 54 |
| *plt9-2* | 45 | 48 | 54 |
| *plt9* null sister | 48 | 50 | 56 |
| *ap2_22-1* | 46 | 53 | 56 |
| *ap2_22-2* | 45 | 46 | 52 |
| *Ap2_22* null sister | 52 | 54 | 61 |
